# Thermoregulatory, metabolic, and cardiovascular responses during 88 min of full‐body ice immersion – A case study

**DOI:** 10.14814/phy2.14304

**Published:** 2019-12-27

**Authors:** Coen C. W. G. Bongers, Thijs M. H. Eijsvogels, Dick H. J. Thijssen, Maria T. E. Hopman

**Affiliations:** ^1^ Radboud Institute for Health Sciences Department of Physiology Radboud University Medical Center Nijmegen The Netherlands; ^2^ Research Institute for Sport and Exercise Science Liverpool John Moores University Liverpool United Kingdom

**Keywords:** cold exposure, G‐Tummo meditation, hypothermia, thermoregulation

## Abstract

Exposure to extreme cold environments is potentially life‐threatening. However, the world record holder of full‐body ice immersion has repeatedly demonstrated an extraordinary tolerance to extreme cold. We aimed to explore thermoregulatory, metabolic, and cardiovascular responses during 88 min of full‐body ice immersion. We continuously measured gastrointestinal temperature (T_gi_), skin temperature (Tskin), blood pressure, and heart rate (HR). Oxygen consumption (VO_2_) was measured at rest, and after 45 and 88 min of ice immersion, in order to calculate the metabolic heat production. Tskin dropped significantly (28–34°C to 4–15°C) and VO_2_ doubled (5.7–11.3 ml kg^−1^ min^−1^), whereas Tgi (37.6°C), HR (72 bpm), and mean arterial pressure (106 mmHg) remained stable during the first 30 min of cold exposure. During the remaining of the trial, Tskin and VO_2_ remained stable, while Tgi gradually declined to 37.0°C and HR and mean arterial blood pressure increased to maximum values of 101 bpm and 115 mmHg, respectively. Metabolic heat production in rest was 169 W and increased to 321 W and 314 W after 45 and 80 min of ice immersion. Eighty‐eight minutes of full‐body ice immersion resulted in minor changes of Tgi and cardiovascular responses, while Tskin and VO_2_ changed markedly. These findings may suggest that our participant can optimize his thermoregulatory, metabolic, and cardiovascular responses to challenge extreme cold exposure.

## Graphical abstract

We investigated the thermoregulatory, metabolic, and cardiovascular responses of the world record holder of full‐body ice immersion when exposed to 88 min of ice immersion. Ice immersion resulted in minor changes of Tgi and cardiovascular responses, while Tskin and VO2 changed markedly.

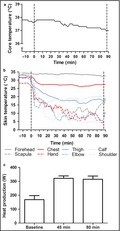

## 1 INTRODUCTION

Hypothermia, defined as a core body temperature below 35°C, is associated with life‐threatening complications, as has been dramatically illustrated by polar expeditions (i.e., Scott's Terra Nova Expedition), ship wrecks (i.e., Titanic), and military operations under extreme cold (i.e., World War II Eastern Front) (Shetty, 2003; Wissler, 2003). Furthermore, athletes competing in colder climates are at risk to develop hypothermia as well (Melau, Mathiassen, Stensrud, Tipton, & Hisdal, 2019). Even nowadays, hypothermia is still estimated to result in ~1,500 deaths annually in the USA (Brown, Brugger, Boyd, and Paal (2012)). In marked contrast, the world record holder of full‐body ice immersion has repeatedly demonstrated an extraordinary tolerance to extreme cold when exposed to full‐body ice immersion for more than 1 hr. His physiological responses during these cold challenges are unknown, but may reveal new information about regulatory mechanisms that allow for the extraordinary ability to sustain prolonged exposure to extreme cold.

The purpose of this case study was to examine the thermoregulatory, cardiovascular, and metabolic responses of the world record holder of full‐body ice immersion during prolonged full‐body contact with crushed ice. We expected to observe a drop in Tgi and concomitant drop in skin temperature (to minimize heat loss) and an elevation of the metabolic rate (e.g., shivering) to increase heat production (Sessler, 2009).

## 2 CASE PRESENTATION AND METHODS

The world record holder of full‐body ice immersion is a Caucasian male (50 year), with a height of 183 cm, and a body mass of 85 kg (BMI = 25.4 kg/m^2^ and BSA = 2.07 m^2^). He was positioned in an open‐top container filled with crushed ice to the shoulder level (2.45 p.m.). The participant was wearing shorts only, whereas his body was in direct contact with the crushed ice. Core temperature (gastrointestinal temperature; Tgi), skin temperature (Tskin), blood pressure, and heart rate (HR) were measured continuously before, during and after ice exposure. Oxygen consumption was used to determine metabolic activity at rest and after 45 and 88 min of crushed ice immersion. According to prior mutual agreement, ice immersion was finished after 88 min. Signed written informed consent was obtained prior to the study. Ethical approval was waived as ice exposure was part of a demonstration session of the participant, so no additional burden was present. All measurements were conducted in line with the ethical standards of the Declaration of Helsinki.

The participant performed Asian G‐Tummo meditation, 1 hr before and during the experiment. G‐Tummo meditation consists of both somatic and neurocognitive components. The somatic component involves specialized breathing techniques, defined as “the vase,” that are accompanied with isometric muscle contractions. Moreover after inhalation that practitioners are holding their breath during which they contract both the abdominal and pelvic muscles (lower belly is shaping like a vase) (Evans‐Wentz, 1958; Kozhevnikov, Elliott, Shephard, & Gramann, 2013). The neurocognitive component consists of meditative visualization that requires the generation and maintenance of mental images of flames at specific locations in the body accompanied by intense sensations of bodily heat in the spine (Evans‐Wentz, 1958; Kozhevnikov et al., 2013).

### 2.1 Thermoregulatory responses

Tgi was measured continuously (every 20 s and averaged per minute) using a telemetric temperature capsule (CorTemp system, HQ Inc) and an external recorder, which has been demonstrated to be safe and reliable (Byrne & Lim, 2007; Gant, Atkinson, & Williams, 2006). The participant ingested an individually calibrated capsule 5 hr prior to the experiment, to ensure passage through the stomach (Wilkinson, Carter, Richmond, Blacker, & Rayson, 2008).

Tskin was measured continuously (every 20 s and averaged per minute) using wireless temperature recorders (iButton DS1922L, Dallas Semiconductor Corp) (Marken Lichtenbelt et al., 2006; Smith et al., 2009). The temperature recorders were attached to the skin using Tegaderm Film (Tegaderm). Tskin was measured at eight distinct locations (forehead, chest, scapula, shoulder, elbow, hand, thigh, and calf), and a weighted average Tskin per minute was calculated according to the ISO‐9886 norm (2004).

### 2.2 Cardiovascular responses

Responses in HR and arterial blood pressure were continuously measured during the first 80 min of ice immersion using an automated blood pressure monitoring device attached to the third digit of the left hand at a sampling rate of 100 Hz (Nexfin, BMEYE). A pulse oximeter (Nellcor N‐200 E, Nellcor) and a manual sphygmomanometer were used every 10 min as a control for the continuous Nexfin measurements.

### 2.3 Metabolic responses

Oxygen consumption (VO_2_) and carbon dioxide (VCO_2_) production were measured by indirect calorimetry using a calibrated gas exchange analyzer (Quark CPET, Cosmed) with a breathing mask. The mask was carefully placed over the mouth and nose to avoid of air leakage. Metabolic heat production (M in Watt) was subsequently calculated using the following formula:

(1) $$M={\text{VO}}_{2}\frac{\left(\frac{\text{RER}-0.7}{0.3}\right)*\text{Ec}+\left(\frac{1.0-\text{RER}}{0.3}\right)*\text{Ef}}{60}*1000$$

where VO_2_ is the oxygen consumption in L/min, RER is the respiratory exchange ratio (VCO_2_/VO_2_), and Ec and Ef are the caloric equivalents per liter of oxygen for the oxidation of carbohydrates (21.13 kJ) and fats (19.62 kJ), respectively (Murgatroyd, Shetty, & Prentice, 1993). During the test, VO_2_ and metabolic heat production were measured at baseline, after 45 min and after 80 min of ice immersion, and averaged over a 3‐min recording.

## 3 RESULTS

### 3.1 Thermoregulatory responses

**Figure 1:**
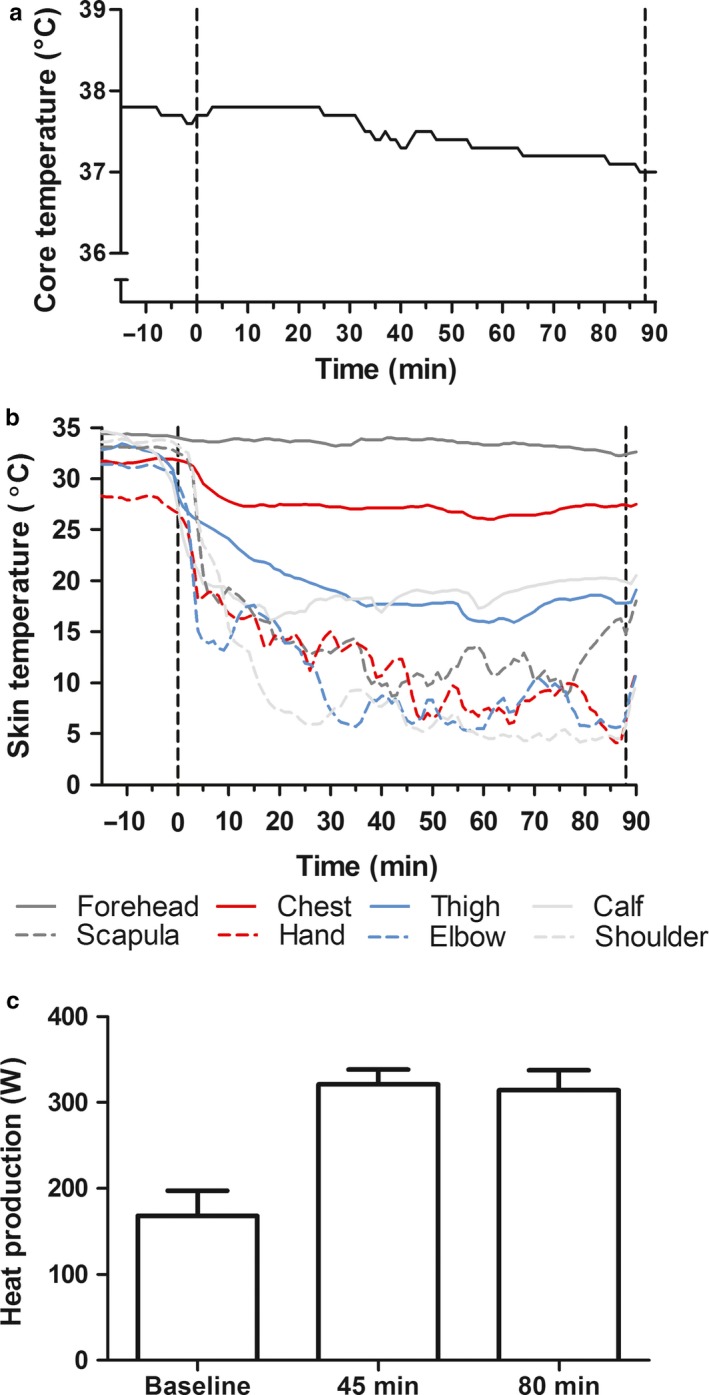
(a) Tgi before, during, and after ice immersion. Vertical dotted lines delineate the start (*t* = 0 min) and end (*t* = 88 min) of ice immersion. (b) Tskin before, during, and after ice immersion. Vertical dotted lines delineate the start (*t* = 0 min) and end (*t* = 88 min) of ice immersion. (c) Metabolic heat production before (baseline) and during (45 and 88 min) ice immersion

Temperature of the crushed ice was 0.14 ± 0.06°C. Tgi during the 10 min before ice immersion was stable at 37.7 ± 0.1°C. Tgi did not change during the first 32 min of full‐body contact with ice (Figure 1a). During the remaining 56 min, Tgi gradually decreased to 37.0°C.

Tskin of the forehead (not in contact with ice) demonstrated a gradual decrease from 34°C (pre‐immersion) to 32°C at the end of ice immersion (Figure 1b). Tskin of the upper chest (measured just above ice level) started at 32°C (pre‐immersion) and reached a plateau at 26°C after 10 min of ice immersion (Figure 1b). Tskin of the shoulder, elbow, hand, and scapula (i.e., body areas which are in direct contact with ice) rapidly decreased from 28–34°C (pre‐immersion) to temperatures as low as 4–15°C within 20 min of ice immersion, and remained stable at this level until the end of immersion. The thigh and calf, also areas in direct contact with ice, demonstrated a drop in Tskin from 33–35°C (pre‐immersion) to 18–20°C during ice immersion. The decrease observed in the thigh and calf was slightly less than that of parts of the upper body that were in contact with the ice.

### 3.2 Cardiovascular responses

**Figure 2:**
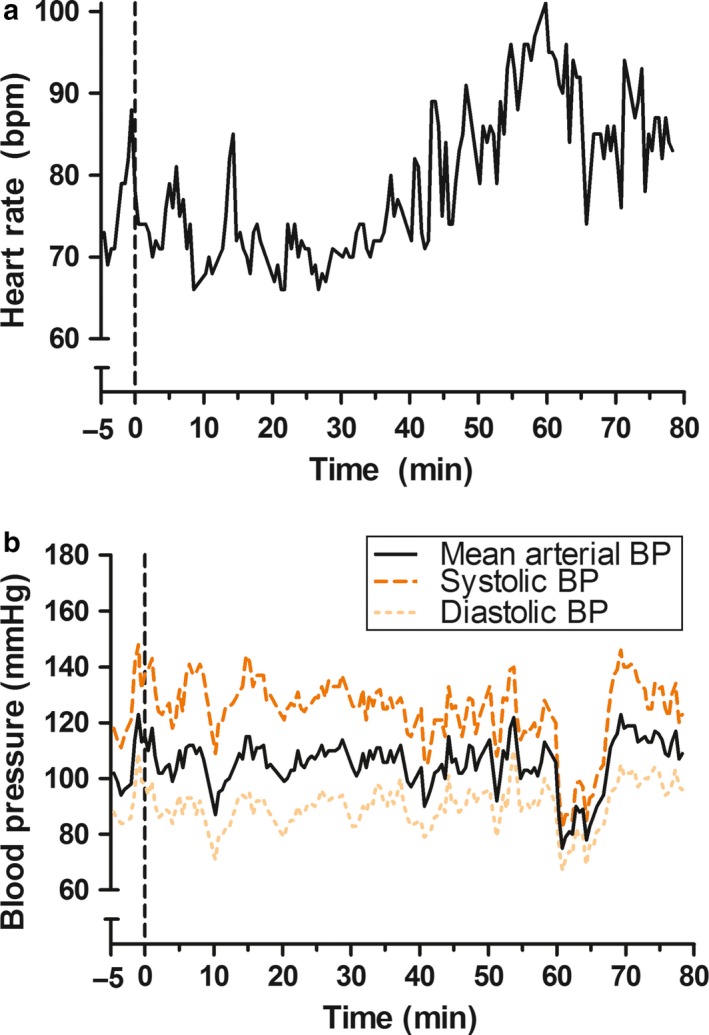
(a) HR before and during ice immersion. A vertical dotted line delineates the start (*t* = 0 min) of ice immersion, while measurements were terminated after 80 min of recording. Bpm, beats per minute. (b) Blood pressure responses before and during ice immersion. Systolic, diastolic and mean arterial blood pressure are presented. A vertical dotted line delineates the start (*t* = 0 min) of ice immersion, while measurements were terminated after 80 min of recording. The transient (5 min) drop in blood pressure after 60 min is likely due to technical problems related to the start of a new recording

Resting HR before ice immersion was 77 bpm (range: 69–88 bpm) and HR remained relatively stable at 72 bpm (range: 66–85 bpm) during the first 40 min of ice immersion (Figure 2a). HR started to increase gradually during the last 40 min of ice immersion, reaching a maximum of 101 bpm. Mean arterial blood pressure at rest was 106 mmHg (94–123 mmHg) and remained stable during the first 60 min at 106 mmHg (87–123 mmHg) (Figure 2b). Mean arterial blood pressure increased to 115 mmHg (107–123 mmHg) during the last 10 min of ice immersion (70–80 min). The changes in HR and blood pressure across the ice immersion were confirmed by data obtained from a pulse oximeter and manual sphygmomanometer every 10 min, respectively, which suggested that our continuous heart rate and mean arterial blood pressure measurements were reliable.

### 3.3 Metabolic responses

Oxygen consumption increased significantly from 0.49 ± 0.08 L/min at rest to 0.96 ± 0.05 L/min and 0.93 ± 0.07 L/min after 45 and 80 min of ice immersion, respectively. Accordingly, metabolic heat production at baseline was 169 W and increased to 321 W and 312 W after 45 and 80 min of ice immersion (Figure 1c). No shivering or bodily movement was observed by the researchers or reported by the participant.

## 4 DISCUSSION

In this study, we report that our participant maintained a stable Tgi, HR, and blood pressure during the first part of crushed ice immersion (i.e., 30‐min). In the second part of crushed ice immersion, our participant was still able to maintain a Tgi above 37°C, whereas HR and blood pressure were only modestly increased. There was, however, a drop in Tskin immediately upon full‐body ice immersion. Despite 88 min of full‐body contact with ice, clinically relevant complications or signs of (thermal) discomfort were not observed. Our study describes the physiological responses which counteract an extreme cold exposure in humans.

Tgi dropped from 37.7 to 37.0°C after 88 min of crushed ice immersion. The decrease of 0.7°C is modest compared to previous studies that reported the onset of hypothermia after ~30 min of cold water immersion (Giesbrecht, 2000; Goheen et al., 1997; Holmer, 1995; Tikuisis, 1997). Furthermore, swimming exercise on a self‐selected pace for 80 min in water at 10°C results in decrease in rectal temperature of 2.6°C/h, in which five out of 10 swimmers did not complete the 90 min (Tipton, Eglin, Gennser, & Golden, 1999). Moreover metabolic heat production in the swimmers was more than twofold higher than the heat production in our participant. Additionally, a recent study demonstrated a drop in gastrointestinal temperature of 1.4°C/h in triathletes swimming in 10°C water while wearing a wetsuit (Melau et al., 2019), which was in line with the study by Tipton and colleagues demonstrating that rectal temperature decreased with 1.6°C/h and 1.1°C/h while swimming without wetsuit in 16°C and 18°C water, respectively (Tipton et al., 1999). A study by Wakabayashi, Hanai, Yokoyama, & Nomura (2006 demonstrated that the Tgi of healthy Japanese male participants (BSA = 1.79 m^2^) decreased with 1.1 ± 0.1°C after 60 min of water immersion at 26°C in normal swim suit. Although clear differences exist between ice immersion and cold‐water immersion (e.g., conductive capacity of water vs. ice), the ability to sustain the cold tolerance and maintain a relatively stable Tgi is remarkable. One explanation for this finding may be the ~2‐fold increase in metabolic heat production in the absence of shivering or any other voluntary bodily movement. The level of oxygen consumption achieved during ice immersion is, in daily living and under normal conditions, comparable to light intensity exercise such as walking (Cramer & Jay, 2016). The increased heat production and oxygen consumption during ice immersion might partly be due to the activity of brown adipose tissue, which has been suggested to be responsible for non‐shivering thermogenesis (Marken Lichtenbelt et al., 2009; Nedergaard & Cannon, 2010). However, in a previous study using the same participant and his monozygotic twin brother (who was not cold acclimatized), cold air exposure (13°C) did not elicit differences in brown fat activity (Vosselman, Vijgen, Kingma, Brans, & Marken Lichtenbelt, 2014). Therefore, it is not likely that the increased heat production (~150 W) during crushed ice immersion can be explained by brown fat activity in our participant.

Before and during ice immersion, our participant performed Asian G‐Tummo meditation to enhance metabolism and maintain Tgi. Previous studies indicated that this meditation technique is associated with an increased metabolism and core temperature (Benson et al., 1982; Kozhevnikov et al., 2013). As our participant performed Asian G‐Tummo meditation prior to ice immersion as well, his metabolic heat production in rest was higher, which could explain the relatively high baseline Tgi. However, Tgi was still within reference values for resting core body temperature (Wunderlich, 1868). We were unable to perform a control experiment in which he was exposed to the same stimulus without meditation. Therefore, it remains speculative whether such meditation represents the principle explanation for our results.

Repeated exposure to extreme cold, that is, cold training, may have activated and/or enhanced metabolic‐insulative body responses. One such mechanisms relate to an enhanced vasoconstriction capacity (Makinen, 2010). Peripheral vasoconstriction is a powerful mechanism to reduce heat loss, but may also cause cold injury due to prolonged low skin temperatures. Previous studies demonstrated that blood vessels typically dilate after 5 to 10 min of cold exposure, resulting in an increase in Tskin (Cheung & Daanen, 2012). This cold induced vasodilation is followed by a new phase of vasoconstriction, which is known as the “hunting reaction” of Lewis (1941), and is observed in the hand, feet and face (Brajkovic & Ducharme, 2006; Daanen, 2003; Greenfield, Kernohan, Marshall, Shepherd, & Whelan, 1951). Previous studies indicated that these local responses are more pronounced in individuals regularly exposed to cold (e.g., individuals from polar tribes, fisherman, fish filleters, etc.) (Daanen, 2003) and with cold acclimation (Livingstone, Nolan, & Keefe, 1993; Purkayastha, Ilavazhagan, Ray, & Selvamurthy, 1993). Whether the cyclic process of alternating cutaneous vasoconstriction and vasodilation “trains” the human body to prevent tissue damage is currently unknown. Interestingly, after cessation of the experiment, the skin displayed a generalized characteristic pattern of the “hunting reaction” of Lewis. Additionally, we were not able to draw a venous blood sample at the final stage of ice immersion, suggesting severe vasoconstriction. Therefore, our results suggest that our participant may have optimized his physiological response to cold stress to effectively counteract the cold stimulus through profound (peripheral) vasoconstriction and a higher metabolism that could have contributed to the cold tolerance.

Our study has some limitations. First, we describe the physiological responses of 88 min of ice immersion in a single participant, making it impossible to extrapolate our findings to the general population. Nevertheless, case reports with remarkable findings can yield valuable information and are important in hypothesis generation for further research. This study is further limited by the absence of a control condition without G‐Tummo meditation. A randomised controlled intervention study should be performed to examine whether G‐Tummo medication is able to alter these responses in participants who are a priori not used to ice immersion. Another limitation of our study, is that we visually checked whether our participant demonstrated shivering thermogenesis, whereas it would have been better to quantify shivering thermogenesis by performing EMG measurements in order to examine the presence of low‐level shivering or an increased muscle tone. Furthermore, we did not measure the percentage of body fat of our participant, which could have influenced our results, as a higher body fatness is associated with a higher insulative capacity and thereby a lower heat loss in cold environments. Additionally, it has been demonstrated that heat production increases during cold exposure, due to the development of electrical activity in the “resting muscles,” defined as thermoregulatory muscle tone, that occurs before the onset of shivering (Burton, 1937). Unfortunately, we did not measure muscle tone using EMG signals in our experiment, and we cannot establish whether a higher muscle tone can explain the increased heat production in our participant. Finally, our results were not corrected for peripheral vasoconstriction. However, as the hands were not in direct contact with ice and the fact that we did not experience problems with taking these measurements, it is likely that peripheral vasoconstriction did not importantly affect our outcomes and main conclusions.

In conclusion, our participant tolerated 88 min of full body crushed ice immersion, during which Tgi did not drop more than 0.7°C, while no shivering was observed. Potentially, he optimized his physiological responses to effectively counteract the cold stimulus through profound (peripheral) vasoconstriction and marked elevation of metabolism.

## CONFLICT OF INTEREST

The authors report no conflict of interest.
